# Tooth Mg/Ca ratios and Aristotle's lantern morphometrics reflect trophic types in echinoids

**DOI:** 10.1002/ece3.11251

**Published:** 2024-06-09

**Authors:** Dimitris Vafidis, Anastasios Varkoulis, Stefanos Zaoutsos, Konstantinos Voulgaris

**Affiliations:** ^1^ Department of Ichthyology and Aquatic Environment Nea Ionia, University of Thessaly Volos Greece; ^2^ Department of Energy Systems University of Thessaly Larisa Greece

**Keywords:** Aristotle's lantern, echinoid invasion, HCPC, Mediterranean, skeletal Mg/Ca ratio

## Abstract

Several studies have inferred the ecological significance regarding the morphometrics of Aristotle's lantern and the mechanical properties of magnesium in echinoid teeth. This study attempts to combine these aspects, connecting them to the trophic habits of three native and an invasive echinoid in the Eastern Mediterranean Sea. Spatiotemporal data from the central and southern Aegean Sea were obtained, regarding the relative size of lanterns and demi‐pyramids of *Arbacia lixula, Paracentrotus lividus*, *Sphaerechinus granularis*, and the invasive echinoid *Diadema setosum* and the Mg/Ca ratios of four zones on the tooth cross‐section. Since environmental factors affect the examined factors, data for temperature, salinity, and concentration of chlorophyll‐a were included in a principal component analysis. *A. lixula* and *P. lividus* presented intraspecific differences in the relative size of the lantern and demi‐pyramid, while *S. granularis* and *D. setosum* exhibited variation in the elongation index. Differences in the Mg/Ca ratios were observed for all species although in different zones. Temperature appears to be related to all Mg/Ca zones except for the stone part, while the elongation index showed an inverse trend to all other morphometric parameters. The results of the PCA for the four species on the spatiotemporal level exhibited a distinction of individuals with season but not species, except for *A. lixula*, an omnivore with a carnivorous tendency, which was clearly separated from the herbivorous species. Using hierarchical clustering on the principal components it was evident that the three native species occupy different clusters, but when *D. setosum* was added, it shared the same cluster with *S. granularis*. This might infer similar feeding preferences, specifically for coralline algae, which might lead to a swift in the ecological equilibrium in regions, where *D. setosum* is found, either by affecting habitat type, or by restricting the distribution of *S. granularis* as was previously observed with *Diadema africanum*.

## Graphical abstract

Tooth morphometrics and mineralogy infer inter‐ and intraspecific differences in feeding habits. Abiotic factors might regulate this relationship.

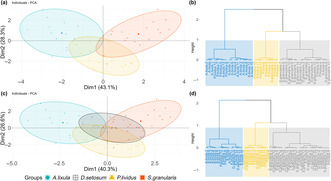

## 1 INTRODUCTION

The feeding ecology of a species influences its population dynamics, affecting key aspects such as resource partitioning, habitat preference, prey selection, and competition and trophic ecology. The feeding behavior of echinoids has long been a subject of interest. Most sea urchins are primarily herbivorous (Johnson & Mann, 1982). They exhibit a variety of feeding habits and behaviors with seasonal and spatial variations in their feeding activity (e.g., De Riddler & Lawrence, 1982).

Plasticity is crucial for an organism not only to survive but also to thrive under changes in food availability (Levitan, 1991). Sea urchins are observed to utilize energy allocation in different organs depending on the availability of resources (Hill & Lawrence, 2003). The relative length of the demi‐pyramid to test diameter is an adaptive morphological characteristic which increases when food is scarce (Ebert, 1980; Ebert et al., 2014; Levitan, 1991). However, this is not necessarily a plastic response to food scarcity but might be related to different growth rates due to variation in food availability, as shown by deVries et al. (2019). Hagen (2008) proposed that the enlargement of the lantern is a functional specialization for durophagy. The plasticity of the lantern of *Arbacia dufresnii* populations with different feeding habits is not dependent on a trophic source but could indicate different trophic tendencies among species (Agnetta et al., 2013; Bonaviri et al., 2011; Epherra et al., 2015). Thus, it is possible that different species exhibit different morphometric profiles depending on their feeding habits, which they might alter either when food is scarce or when they change the source of food intake.

The self‐sharpening mechanism of the sea urchin tooth has recently drawn much attention (e.g., Espinosa et al., 2019; Killian et al., 2011; Ma et al., 2009). The tooth cross‐section of echinoids presents a variety of shapes, from keeled and grooved to wedge‐like and diamond‐shaped, which is hypothesized to confer different functional properties (Ziegler et al., 2012). The Mg content of echinoid teeth seems to be related to hardness, however, it is shown to vary with skeletal element, taxonomy, and latitude, while abiotic factors like temperature, salinity, and ambient seawater Mg/Ca ratio and even food source also seem to have a strong influence on the mineralogy of the echinoid skeleton, which would mean that the mechanical properties of the tooth can vary not only among species, but also between intraspecific populations (Kolbuk et al., 2019; Ma et al., 2009; Smith et al., 2016; Varkoulis et al., 2020).

When viewed in cross‐section, the mature part of the sea urchin tooth exhibits three different sections, namely, the primary plates, the stone part, and the keel. The primary plates section is composed of layers of plates parallel to each other, while the stone part is constructed by calcareous needles connected to the primary plates. Finally, the major building structures of the keel are called prisms. It has been shown that hardness and Mg content decrease, as the distance from the stone part (the hardest section of the tooth) increases (Markel & Gorny, 1973; Wang et al., 1997). However, a clear relationship between abiotic factors and taxonomy with hardness or mineralogy of specific sections of the tooth has yet to be established.

The recent thermal increase in the Mediterranean Sea has intensified the introduction of invasive tropical species (Bianchi, 2007). The first record of an invasive echinoid in the Mediterranean was that of *Diadema setosum*, which was reported in 2006 in Kas peninsula and since then its distribution has been extended in the Aegean sea, where established populations have been reported (Vafidis et al., 2021; Yokes & Galil, 2006). A successful invasion can only take place, when the invading species can cope with the new environmental conditions and usually exhibits some adaptive advantage (Smith, 2009). In the case of *D. setosum* it was previously shown that it presented decreased Mg content in the skeleton compared to its counterparts in the Pacific without compromising the survival rate due to its chemical defense (Voulgaris et al., 2021). However, there is neither evidence regarding the interactions of *D. setosum* with the native echinoid species of the shallow sublittoral zone, namely *Arbacia lixula*, *Paracentrotus lividus*, and *Sphaerechinus granularis* nor information about its feeding ecology in the Mediterranean.

This study compares the morphometric indices of the Aristotle's lantern and demi‐pyramids, while also examining variations in the Mg/Ca ratios of the tooth of four echinoid species with different dietary preferences, to infer whether there is a connection among diet, morphometrics, and mineralogy in echinoids. Subsequently, the trophic role of the invasive echinoid *D. setosum* in the Eastern Mediterranean Sea is speculated.

## 2 MATERIALS AND METHODS

### 2.1 Study area and satellite remote sensing data

**FIGURE 1:**
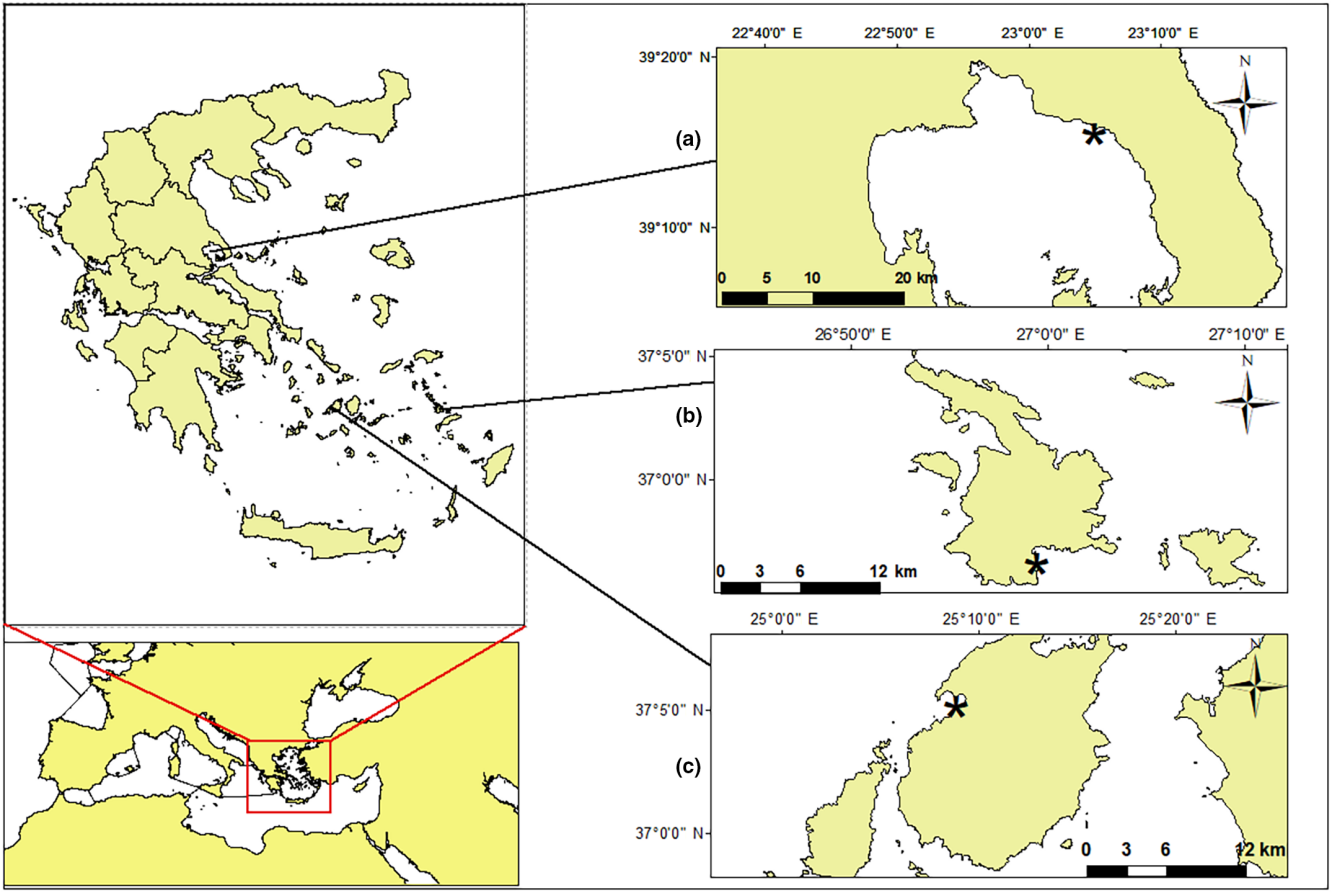
Study sites of (a) Pagasitikos gulf, (b) Dodecanese island complex (Kalymnos), and (c) Cyclades island complex (Paros); * indicates the sampling stations.

This study was carried out at three sites of the Aegean Sea, Eastern Mediterranean, namely at Pagasitikos Gulf (39°18′25.5702″ N, 23°5′53.4222″ E), at the island of Kalymnos, Dodecanese Island complex (36°56′15.5328″ N, 26°59′16.0542″ E), and at the island of Paros, Cyclades Island complex (37°5′30.4686″ N, 25°9′19.6884″ E) (Figure 1). The Aegean Sea is characterized by complicated topography, making it a unique island archipelago, with over 2000 islands, numerous gulfs, embayments, and straits. The gulf of Pagasitikos is located in the central‐east Aegean, whereas both the Dodecanese and Cyclades Island complexes are situated in the southern part of the Aegean. The southern basin is distinguished from its central and northern counterparts by substantial different water masses (Zervakis et al., 2000). This is reflected by differences in both abiotic and biotic factors, with the most prominent parameter being temperature (Androulidakis & Krestenitis, 2022). The two island complexes are separated by deep seas. Furthermore, the Dodecanese complex is considered the gateway for Lessepsian species, which tends to alter the biodiversity in the Mediterranean Sea (Azzurro et al., 2011; Pancucci‐Papadopoulou et al., 2012). All of the aforementioned features regarding the three studied regions could be summarized in which they belong to different biogeographical modules, each with different evolutionary history, as shown in Kougioumoutzis et al. (2016).

To examine the effect of seasonality, samplings were caried out in December (WinPag) and June (SumPag) of 2020 at Pagasitikos Gulf for the three native species and in the same months at Kalymnos Island (WinDod: December in Kalymnos; SumDod: June in Kalymnos) for *D. setosum*. Additional samplings were performed in December in the island of Kalymnos for *A. lixula*, *P. lividus*, and *S. granularis* and in June in the island of Paros (SumCyc) for the invasive species, where a newly established population was found, to assess the spatial effect. Sampling sites and periods were chosen taking into account the size and number of individuals for each species (Figure 1).

Data regarding the sea surface temperature, salinity, and concentration of chlorophyl‐a were acquired from the NASA EOSDIS Physical Oceanography Distributed Active Archive Center (PO.DAAC) (https://podaac.jpl.nasa.gov/) and Ocean Biology Distributed Active Archive Center (https://oceancolor.gsfc.nasa.gov/). Specifically, temperature was obtained from the Group for High Resolution Sea Surface Temperature (GHRSST) SST (version 1.0) product, salinity from the Soil Moisture Active Passive (SMAP) Project, and chlorophyl‐a from the Ocean Biology Processing Group. The GHRSST (K10L4) archived data are produced on an operational basis at the Naval Oceanographic Office (NAVOCEANO) on a global 0.1 degree grid at daily temporal resolution, while SMAP produces data on a global 0.6 degree grid at 8‐day intervals. Finally, the data for chlorophyll‐a were acquired with a resolution of 4 km grids produced from daily datasets. In order to account for daily variation, monthly data were acquired characterizing the abiotic conditions in each region and season.

### 2.2 Sample collection and morphometric measurements

Forty adult individuals of the three native species, namely *P. lividus*, *A. lixula*, and *S. granularis* and of the invasive *D. setosum* were collected at each site and season from the shallow sublittoral zone (1.5–5 m) by SCUBA diving. Previous studies have determined that sea urchins might shift their dietary preferences from juvenile to adult; thus, only adult individuals were collected (González‐Durán et al., 2008). Regarding the native species, adult individuals were considered those with test diameter larger than 50 mm, whereas for *D. setosum* individuals over 70 mm were sampled (Boada et al., 2017; Coppard & Campbell, 2006). Once collected they were transported alive in a cooler in the laboratory for processing.

**FIGURE 2:**
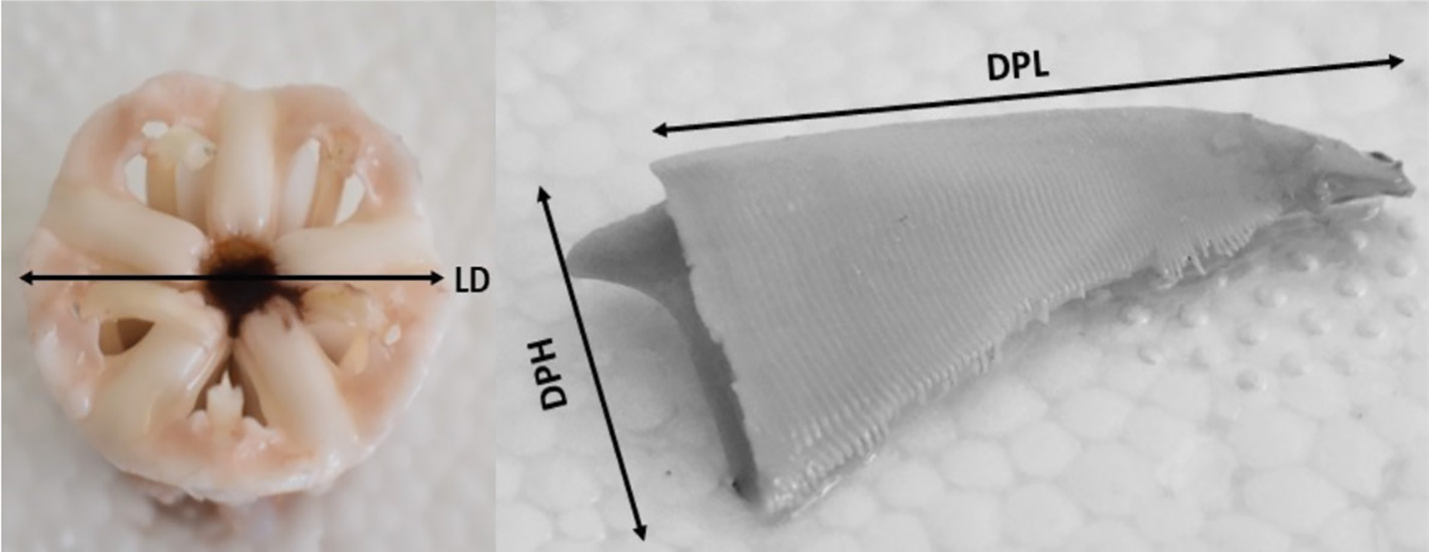
Dorsal view of the Aristotle's lantern with the measurement of its diameter. Longitudinal view of the demi‐pyramid describing the retained measurements of the height and length. DPH, demi‐pyramid height; DPL, demi‐pyramid length; LD, lantern diameter.

The test diameter (D) at ambitus and the height (H) without the spines were measured to the nearest mm using a digital caliper. Each individual was then dissected to acquire the Aristotle's lantern. One demi‐pyramid per individual was haphazardly extracted. Morphometric measurements consisted of the diameter of the Aristotle's lantern (LD), the demi‐pyramid height (DPH), and the demi‐pyramid length (DPL), (Figure 2). Four descriptors/ratios were defined from these parameters and used as proxies for shape namely:

1. Aristotle's Lantern Index (LI) = LD/D,
2. Demi‐pyramid Length to Test Diameter Ratio (DLD) = LP/D,
3. Demi‐pyramid Length to Test Height Ratio (DLH) = LP/H.
4. Demi‐pyramid Elongation Index (DhDl) = DPH/DPL.

### 2.3 Energy dispersive spectroscopy analysis (EDS)

**FIGURE 3:**
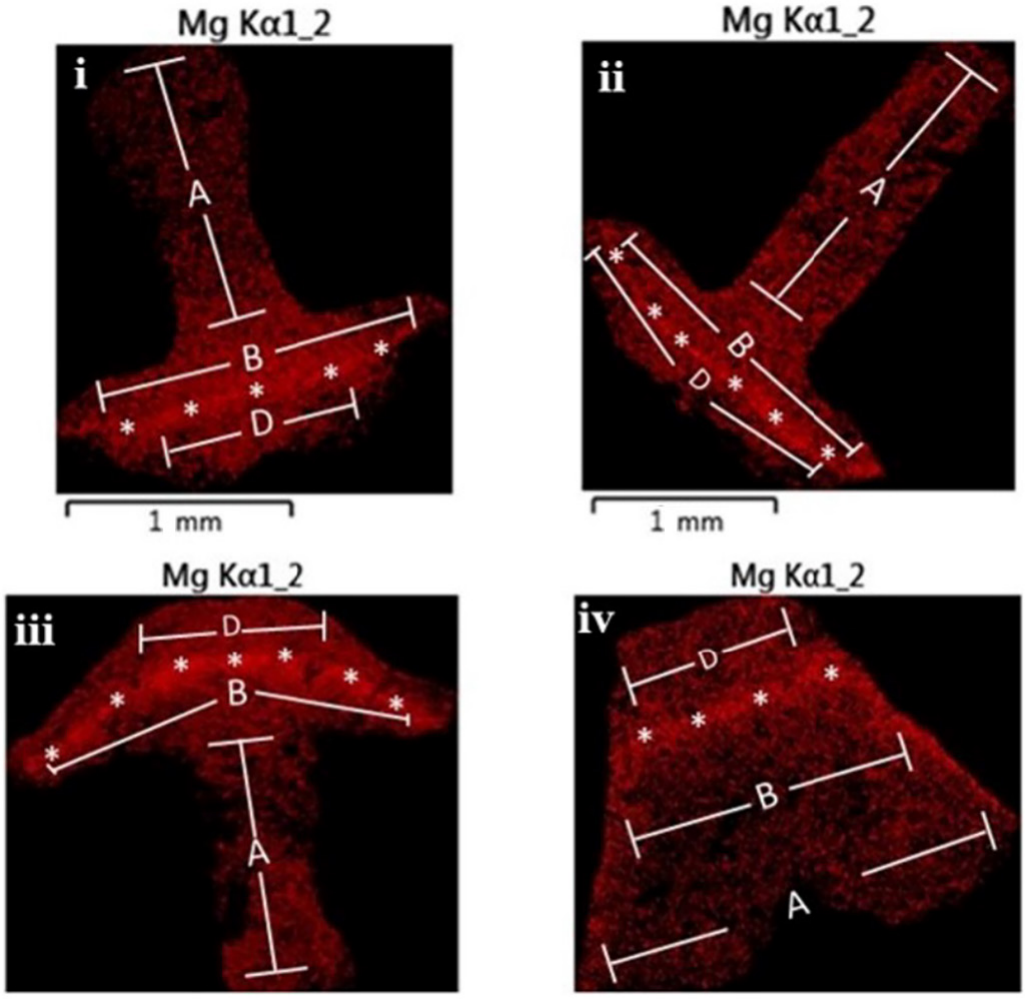
Elemental map of magnesium concentration showing the micromorphology as well as the four distinct zones of the cross section of the tooth. More intensive red indicates higher concentration, (i) *A. lixula*, (ii) *S. granularis*, (iii) *P. lividus*, and (iv) *D. setosum*; * indicates zone C (stone part).

To semi‐quantify the magnesium/calcium (Mg/Ca) ratio in the teeth, 10 individuals per treatment and species were analyzed using a JEOL JSM 6510 equipped with an Oxford Link ISIS 300 system. The teeth were acquired by carefully dissecting the demi‐pyramids. They were subsequently gently rinsed with sodium hypochlorite (5%) followed by fresh water to remove the soft tissue. Further processing consisted of transversally sectioning the teeth with a diamond saw near the tip, where the keel (or the groove for *D. setosum*) was fully exposed, and grinding and polishing the samples from both sides. The processed samples were then mounted in metal stabs with a carbon‐based tape and spatter‐coated with 6 nm carbon (Varkoulis et al., 2020). To determine variations in different parts of sea urchin teeth, four zones were established and stratified by both distance and orientation relative to the stone part as suggested by Masic and Weaver (2015), (Figure 3). A minimum of three measurements per zone was taken from an elliptical area of about 200 μm^2^.

### 2.4 Statistical analysis

One‐way ANOVA was applied to investigate intraspecific spatio‐temporal variations both for morphometric indices and Mg/Ca content. Variations among species and zones were also determined by one way ANOVA. Prior to the analyses, data were tested for normality and homogeneity, using the Kolmogorov and Kohran's test, respectively. Post‐hoc comparisons were carried out using the Tukey's HSD test, which controls for Type I errors resulting in multiple comparisons. One‐way ANOVAs were performed using the software Minitab ver. 19 (Minitab, Inc, 2019).

Additionally, a principal component analysis was conducted for the four morphometric indices and the Mg/Ca ratios of the four zones on the species level, to determine interspecific similarities among the morphometrical and mineralogic profiles. PCA was also employed to examine the combined effect of morphometric indices, Mg/Ca zones, and abiotic factors on each species by treatment. For this purpose, the “FactoMineR”, “factoextra”, and “ggplot2” packages in the R® software (V 4.2.0) were used. Finally, to obtain information about the level of similarity among the species, when the combined effect of morphometrics and chemical composition is taken into account, hierarchical cluster analysis on the principal components (HCPC) was used. Using the “FactoMineR” package, the algorithm of the HCPC method was implemented. Using the Kaiser's criterion, the number of principal components to be used was determined and the HCPC function adopted the Euclidean distance to characterize the distance between observations and the Ward's agglomeration method was used to build the hierarchical tree. To improve the initial partition obtained from hierarchical clustering, K‐means clustering was intergraded in the algorithm (Husson et al., 2010). The visualization of the dendrogram was made with the package “dendextent”.

## 3 RESULTS

### 3.1 Morphometric indices

**FIGURE 4:**
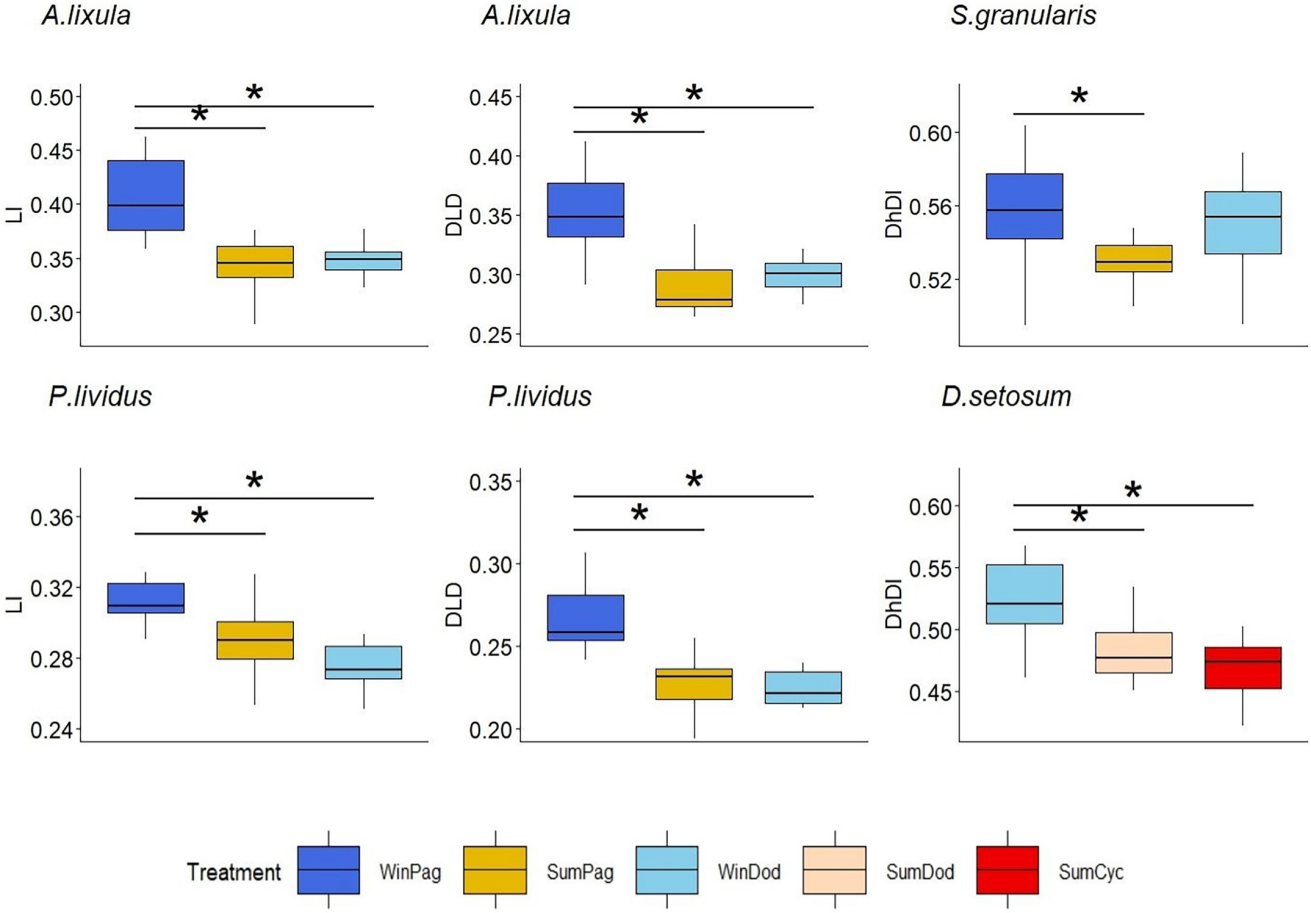
Intraspecific variation for morphometric indices observed in the four examined species. Error bars represent ± standard deviation. Asterisks (*) indicate significant differences among treatments as determined by post‐hoc tests.

On the intraspecific level, two distinct trends were observed, namely either a change in the relative size of the Aristotle's lantern and demi‐pyramid length in relation to test diameter, or a change in the demi‐pyramid elongation index. *P. lividus* and *A. lixula* presented increased values for the lantern index (LI) (*p* = .003; *p* < .0005) and the DLD ratio (*p* < .0005 for both species) in Pagasitikos in winter, compared to individuals from Pagasitikos in summer and from Dodecanese in winter. *S. granularis* and *D. setosum* exhibited significant variation regarding the elongation index (DhDl) (*p* = .036; *p* = .014) (Figure 4). *S. granularis* appeared to have wider demi‐pyramids (DhDl) in Pagasitikos in winter compared to the same region in summer. *D. setosum* exhibited wider demi‐pyramids during winter in Dodecanese, compared to Cyclades in both seasons. Finally, no change was observed in the relative length of the jaw to the test height for any of the examined echinoids.

The examination of the interspecific effect exhibited significant differences for all morphometric ratios (Table 1). The lantern index (LI) of *A. lixula* presented highest values, while *P. lividus* showed the lowest ones. *S. granularis* and *D. setosum* displayed intermediate values. The same pattern was observed for the relative demi‐pyramid length to test diameter (DLD). *S. granularis* exhibited the lowest tooth length to test height (DLH), followed by *P. lividus* and *D. setosum*, whereas *A. lixula* displayed the highest values. Regarding the elongation index (DhDl), *A. lixula* presented lowest values, whereas *S. granularis* exhibited the highest ones. *P. lividus* and *D. setosum* presented intermediate ratios with no statistical differences when compared with each other (Table 1).

**TABLE 1:** One‐way ANOVA and post‐hoc comparison of morphometric indices and Mg/Ca zones with species.

Morphometric indices	*A. lixula*	*D. setosum*	*P. lividus*	*S. granularis*	*p*‐value
LI	0.37 ± 0.04^a^	0.32 ± 0.04^b^	0.29 ± 0.03^c^	0.34 ± 0.03^a,b^	<.001
DLD	0.31 ± 0.04^a^	0.27 ± 0.03^b^	0.24 ± 0.03^c^	0.26 ± 0.02^b^	<.001
DLH	0.59 ± 0.07^a^	0.49 ± 0.06^b^	0.48 ± 0.07^b^	0.43 ± 0.07^c^	<.001
DhDl	0.44 ± 0.02^a^	0.49 ± 0.03^b^	0.49 ± 0.04^b^	0.54 ± 0.03^c^	<.001
Mg/Ca Zones					
A	0.03 ± 0.01^a^	0.04 ± 0.01^a^	0.03 ± 0.01^a^	0.05 ± 0.01^b^	<.001
B	0.06 ± 0.02^a^	0.06 ± 0.01^a^	0.06 ± 0.01^a^	0.08 ± 0.02^b^	<.001
C	0.15 ± 0.02^a^	0.19 ± 0.03^b^	0.16 ± 0.03^a,b^	0.2 ± 0.05^b^	<.001
D	0.06 ± 0.02^a^	0.08 ± 0.02^b^	0.06 ± 0.02^a^	0.09 ± 0.03^b^	<.001
Total	0.08 ± 0.05^a^	0.09 ± 0.06^a,b^	0.08 ± 0.05^a^	0.1 ± 0.06^b^	.003

*Note*: ^a–d^Different letters in the same row indicate significant differences reported by post‐hoc tests (Tukey's test, *p* < .05).

### 3.2 Zoning of Mg/Ca ratios

Interspecific comparisons revealed that *S. granularis* presented the highest Mg/Ca in zone A, while no significant differences were observed among the other three species. The same pattern was noticed for zone B. The stone part (zone C) of *A. lixula* appeared to have the lowest Mg/Ca content followed by *P. lividus*, although the latter did not show any statistical differences with *D. setosum* or *S. granularis*. Regarding zone D, *A. lixula* and *P. lividus* significantly differed when compared to *D. setosum* and *S. granularis*. The total Mg/Ca content of the tooth cross‐section appeared to be the highest for *S. granularis* and the lowest for *A. lixula* and *P. lividus*. *D. setosum* presented intermediate values with no statistical differences with the three native species (Table 1).

**FIGURE 5:**
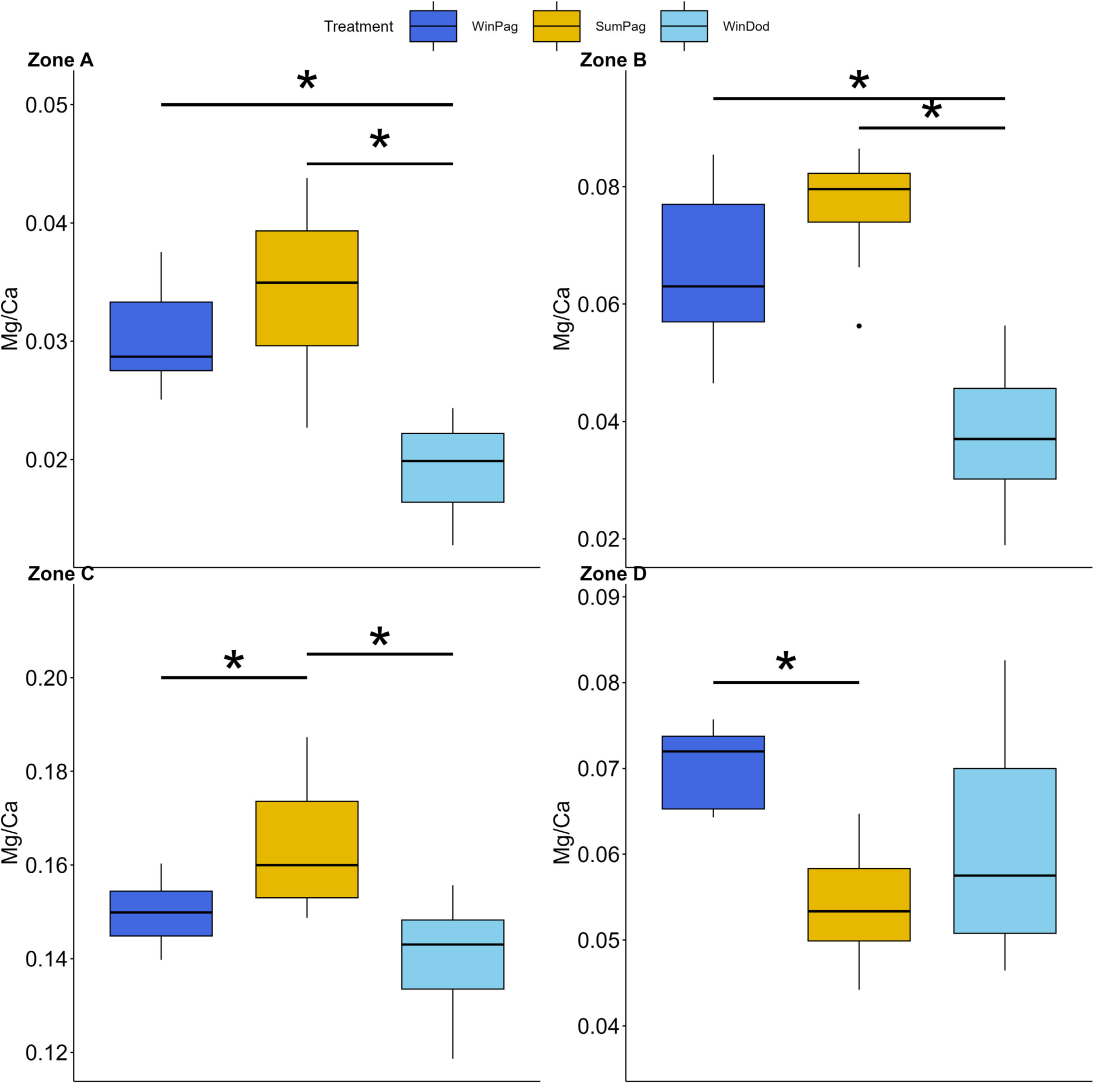
Intraspecific variation in the Mg/Ca ratio for the zones of the cross‐section of the tooth of *A. lixula*. Errorbars represent ± standard deviation. Asterisks (*) indicate significant differences among treatments as determined by post‐hoc tests.

*A. lixula* showed a significantly higher Mg/Ca ratio in Pagasitikos gulf compared to that in the Dodecanese region for zones A and B (*p* < .001 for both zones), while the highest values were observed in summer in Pagasitikos for zone C (*p* = .012). Zone D exhibited a significantly lower Mg/Ca ratio in summer compared to that in winter in Pagasitikos gulf (*p* = .017) (Figure 5).

**FIGURE 6:**
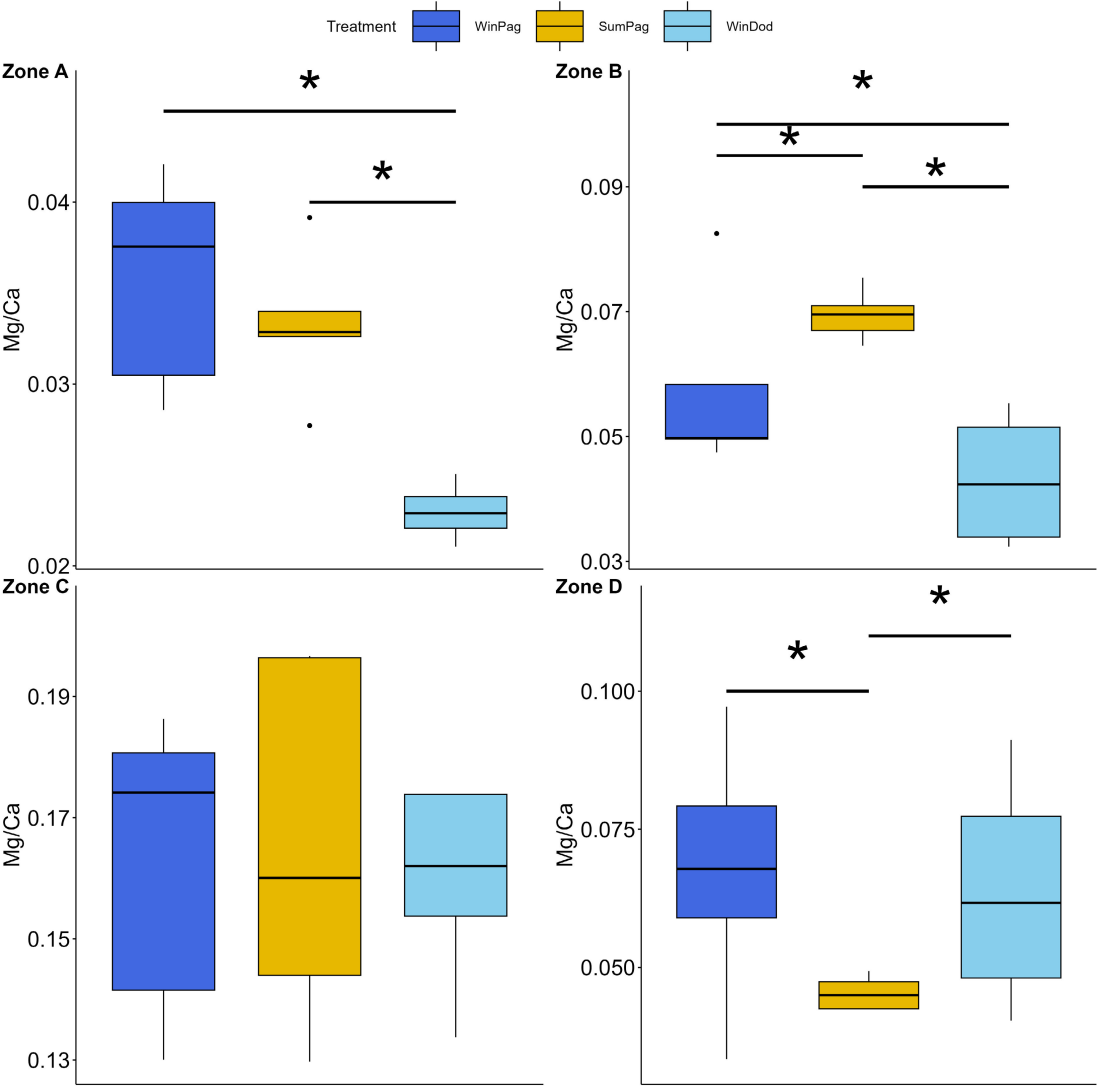
Intraspecific variation in the Mg/Ca ratio for the zones of the cross‐section of the tooth of *P. lividus*. Errorbars represent ± standard deviation. Asterisks (*) indicate significant differences among treatments as determined by post‐hoc tests.

The Mg/Ca ratio of the tooth of *P. lividus* appeared to change between the regions for zones A (*p* < .001) and D (*p* = .003), while zone B (*p* < .001) presented variation among all treatments. Zone C showed no significant differences (Figure 6).

**FIGURE 7:**
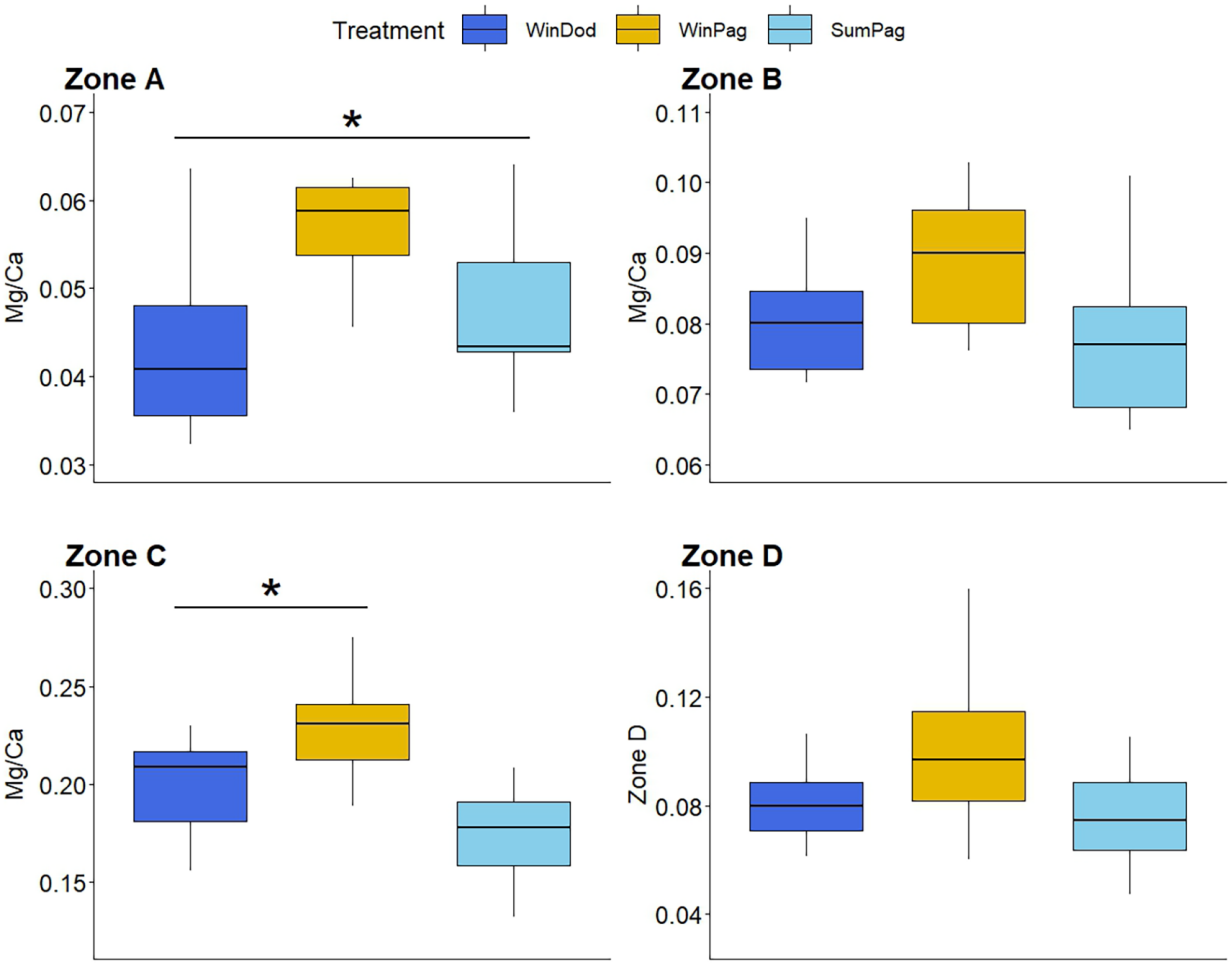
Intraspecific variation in the Mg/Ca ratio for the zones of the cross‐section of the tooth of *S. granularis*. Errorbars represent ± standard deviation. Asterisks (*) indicate significant differences among treatments as determined by post‐hoc tests.

For *S. granularis*, zone C (*p* = .014) exhibited increased values in winter in Pagasitikos gulf compared to that in summer in the same region, whereas for zone A, a higher Mg/Ca ratio was observed in winter in Pagasitikos and it was the lowest in the same season in the Dodecanese region (*p* = .016) (Figure 7). Zones B and D showed no significant differences.

**FIGURE 8:**
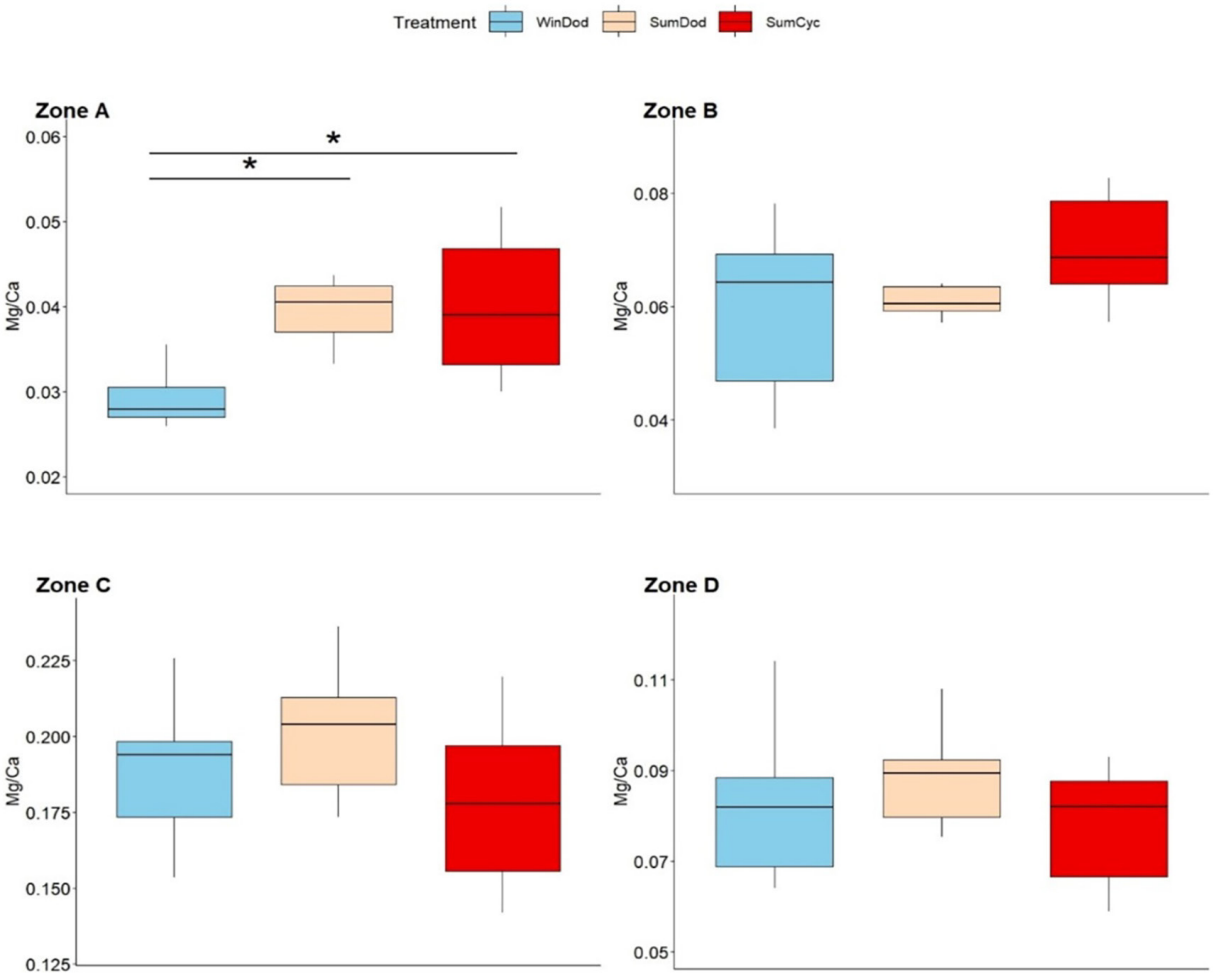
Intraspecific variation in the Mg/Ca ratio for the zones of the cross‐section of the tooth of *D. setosum*. Errorbars represent ± standard deviation. Asterisks (*) indicate significant differences among treatments as determined by post‐hoc tests.

The Mg/Ca ratio of the tooth of *D. setosum* exhibited no significant differences with season or region except for zone A (*p* = .0002), where lower values were observed in winter in the Dodecanese region compared to the other treatments (Figure 8).

### 3.3 Multivariate analysis

**FIGURE 9:**
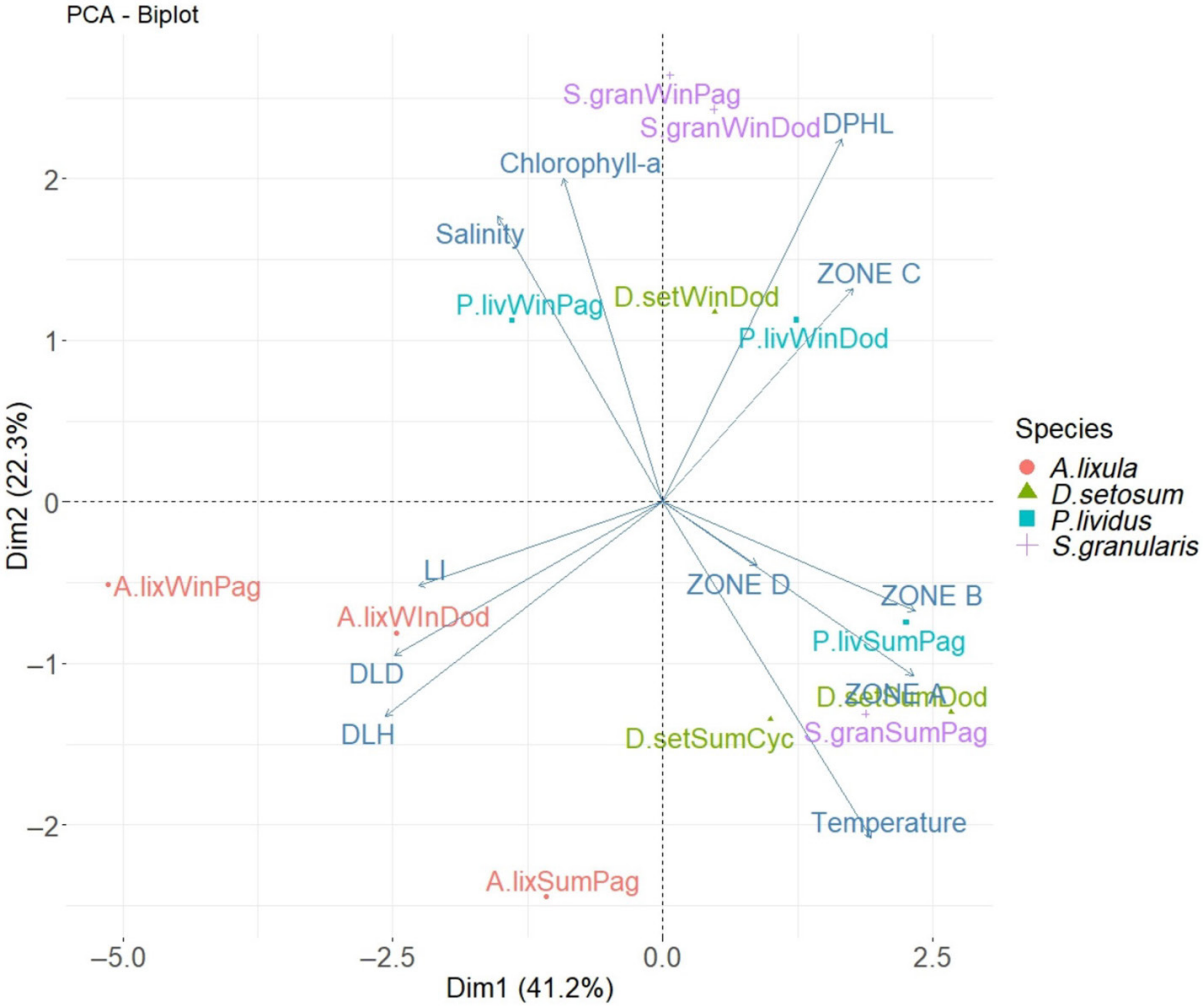
PCA biplot using morphometric indices, Mg/Ca zones, and abiotic variables (blue vectors) of the examined species in each treatment. The lengths of lines correspond to the magnitude of influence of the variable.

The PCA analysis indicates that all examined echinoid species form seasonal clusters except for *A. lixula*, which is separated from the other three species (Figure 9). Zone C was the only Mg/Ca zone, which was not positively correlated with temperature. The elongation index was negatively correlated with the other morphometric indices. Regarding the abiotic factors, salinity and concentration of chlorophyll‐a were negatively correlated with temperature. The three environmental variables showed a low contribution in PC1 and a high contribution in PC2.

**FIGURE 10:**
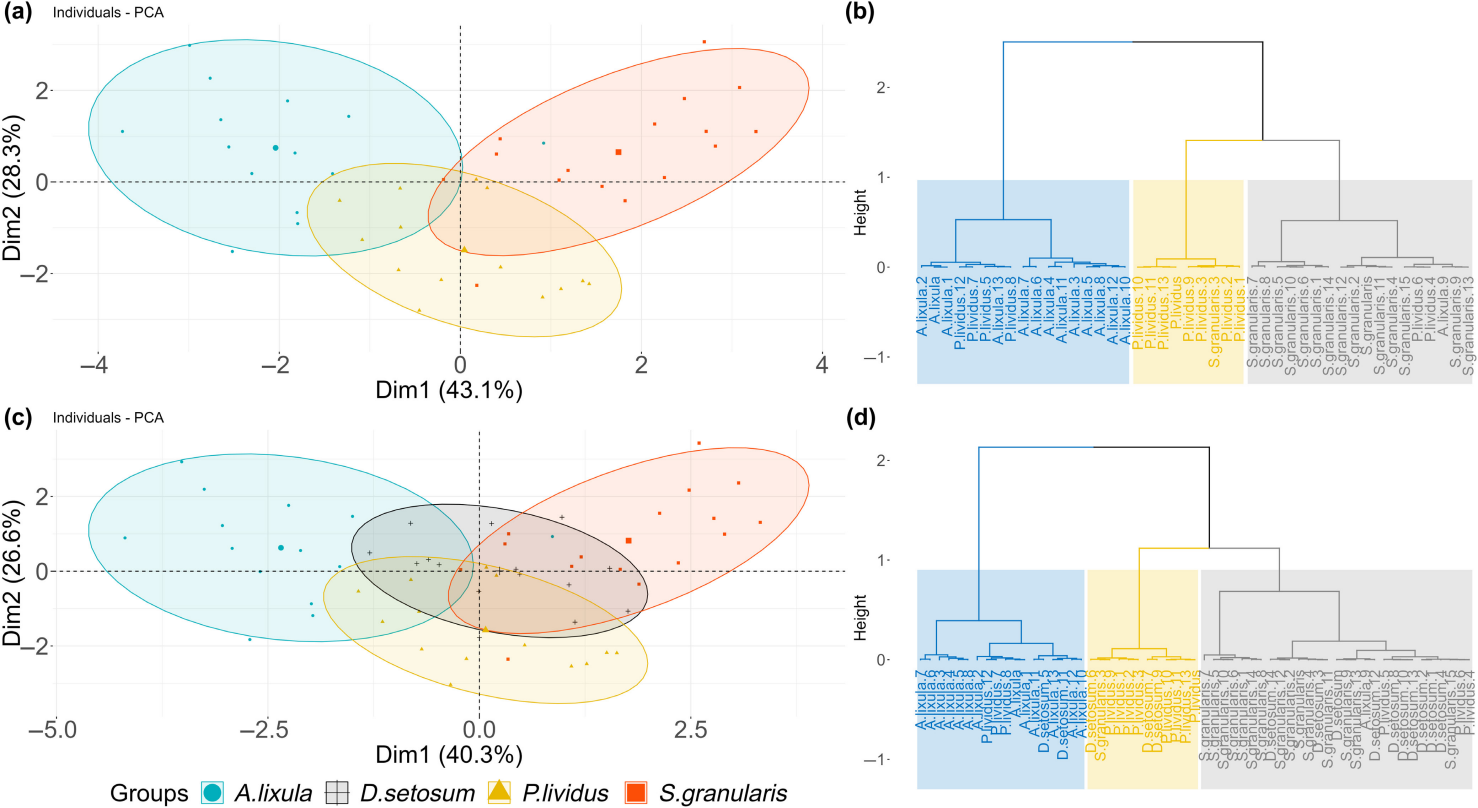
Left: PCA scoreplot for (a) the three native species and (c) the addition of *D. setosum*. Ellipses represent 95% confidence intervals. Right: Dendrograms produced by HCPC depict the resulting clusters for (b) the native species and (d) the addition of *D. setosum*. Different colors indicate different clusters.

Using the Kaiser criterion, the optimal number of clusters to be used for hierarchical clustering on principal components (HCPC) was determined. The first two principal components were used both for the native species and all four species combined, explaining a cumulative variance of 71.4% and 66.9%, respectively. Based on the HCPC two dendrograms were obtained, facilitating the interpretation of the structure of data, both for the native echinoids and the combination of the four species. It is evident, both from the PCA and the HCPC dendrogram, that the three native species are separated from each other (Figure 10a,b). With the addition of *D. setosum*, it becomes evident that this species shares a wide area with the native ones, particularly *S. granularis* and *P. lividus*. The number of clusters remains constant (*n* = 3), while individuals of *D. setosum* are heavily clustered together with the specimen of *S. granularis* (Figure 10c,d). In both dendrograms individuals of *A. lixula* are clustered together and separated from the other two clusters.

## 4 DISCUSSION

It was previously indicated that a functional relationship exists between trophic morphology and diet in regular echinoids (Hagen, 2008). Additionally, the Mg/Ca ratio is hypothesized to be related to increased hardness in the sea urchin tooth (Ma et al., 2007). Thus, a combination of morphological aspects of the Aristotle's lantern and demi‐pyramids combined with the Mg/Ca content of the tooth might have an ecological significance, related to the exploitation of different food sources for different species. The existing literature describes *A. lixula* as an omnivore with a tendency to carnivory, and *P. lividus* as a herbivorous opportunistic generalist with a preference to soft algae, while *S. granularis* and *D. setosum* exhibit herbivory coupled with a tendency towards bioresion (Table 2). Resource allocation might also be affected by feeding preferences, food availability, and environmental conditions (e.g., Ebert, 1980; Ebert et al., 2014; Epherra et al., 2015). Regarding the intraspecific variation of the Mg/Ca ratio in the echinoid tooth the key factors include abiotic conditions, mainly temperature and salinity, as well as diet, although the existing literature is rather limited (Hermans et al., 2010; Kolbuk et al., 2019).

**TABLE 2:** Feeding preferences of the four examined species including their feeding types, as characterized in the literature.

Species	Feeding type	Main food sources	Area	References
*A. lixula*	H	Encrusting corralines	Adriatic Sea (Western Mediterranean)	Privitera et al. (2008)
	O/C	Vegetal items (e.g., Cladophora, Lithophyllum)/Animal items e.g., (Polychaeta, Bryozoa)	NE Spain (Western Mediterranean)	Wangensteen et al. (2011)
	O/C	↑ Lipase (U/mg protein)	Granada Spain (Western Mediterranean)	Trenzado et al. (2012)
	O/C	Vegetal items (e.g., Encrusting algae)/Animal items e.g., (Sipunculids)	Ustica Sicily (Western Mediterranean)	Agnetta et al. (2013)
	C	71% of the guts consisted of *Chthamalus* spp.	São Sebastião (SE Brazil)	Cabral de Oliveira (1991)
*P. lividus*	H	*Posidonia* meadows (leaves and epiflora)	Mediterranean	Verlaque (1981)
	H	Erect fleshy algae	NE Spain (Western Mediterranean)	Privitera et al. (2008)
	O/OG	*Posidonia oceanica*, leaf epiphytes, rhizome algae	NE Spain (Western Mediterranean)	Prado et al. (2010)
	O/H	Mainly vegetal items (e.g., D*ictyota*, *Jania*)/Animal items e.g., (3%–5%)	NE Spain (Western Mediterranean)	Wangensteen et al. (2011)
	H	↑ cellulase activity (preference for flesh algae)	Granada Spain (Western Mediterranean)	Trenzado et al. (2012)
	O/H	Erect fleshy algae	Ustica Sicily (Western Mediterranean)	Agnetta et al. (2013)
*S. granularis*	H	On rocky shore (encrusting coralline red algae)/on *Posidonia* meadows (rhizomes and entire roots)	Mediterranean	Verlaque (1981)
	H/B	Coralline red algae	Bay of Marseilles (Western Mediterranean)	Sartoretto and Francour (1997)
	H	↑ amylase activity (Coralline red algae)	Miguel Island, Azores (N Atlantic)	Trenzado et al. (2012)
	H	Algae	Miguel Island, Azores (N Atlantic)	Hipolito et al. (2020)
*D. setosum*	H/B	Coral sediment (48%–52%)/algae (28%)	Coast of Kenya (Indian Ocean)	McClanahan and Muthiga (1988)
	H	*Codium geppiorum*, *Hydroclathrus clathrus*	Fiji (Pacific Ocean)	Coppard and Cambell (2007)
	B	*Platygyra carnosus*, *Porites lutea*	Hong Kong	Dumont et al. (2013)
	B/C	Scleractinian corals	Hong Kong	Qiu et al. (2014)

Abbreviations: B, bioeroder; C, carnivorous; C, corallivorous; H, herbivorous; OG, opportunistic generalist; O, omnivorous.

The intraspecific comparison of the spatio‐temporal effect on the morphometric indices revealed different trends of variation for different species. *A. lixula* and *P. lividus* alter the size of their mouth appendages in relation to the size of the test, while *S. granularis* and *D. setosum* exhibit variation regarding the elongation index of the teeth. Food availability has long been hypothesized to affect the relative length of the demi‐pyramid and Aristotle's lantern in some echinoid species (e.g., Black et al., 1982; Ebert, 1980; Ebert et al., 2014; except deVries et al., 2019). The results regarding *P. lividus* and *A. lixula* might reflect food scarcity for these species in winter in Pagasitikos gulf, which is also supported by another study (Petihakis et al., 2005) (Figure 4). On the other hand, for *S. granularis* and *D. setosum* a change in width/length of the tooth is observed. This variation follows a seasonal trend, possibly indicating a shift towards a different trophic source, e.g., from erect algae to crustose corraline algae, in order to cope with the seasonal changes in food availability.

Regarding the Mg/Ca content, intraspecific differences were found for all species, although in different zones (Figures 5, 6, 7, 8). Notably, *P. lividus* and *D. setosum* did not alter the Mg/Ca ratio in zone C (stone part), the zone with the most prominent functional role (Markel & Gorny, 1973). Temperature is among the main factors affecting the Mg/Ca content of echinoid teeth (Hermans et al., 2010). *A. lixula* seems to be mainly affected by temperature, while the Mg/Ca content of *S. granularis* seems to follow a similar trend with the elongation index, exhibiting increased values in winter in Pagasitikos (Figure 4). Finally, *D. setosum* exhibits differences only for zone A, which might be related to the different morphology of its grooved teeth compared to the keeled ones (Ziegler et al., 2012). However, a key parameter, which was not examined in this study, is the growth of the tooth, which appears to be continuous, but might be related to the degree of the grind and wear mechanism among different species (Gorzelak et al., 2017; Märkel, 1970).

The multivariate analysis using morphometrics, mineralogical, and environmental data indicates that *A. lixula* is separated from all other species, while for *P. lividus*, *S. granularis*, and *D. setosum* a clear effect of temperature exists, leading to a clustering of treatments rather than species (Figure 9). The fact that *A. lixula* exhibits a different diet compared to the other examined species, might be related to the separation observed here, although no causal connection can be made from this study.

On the interspecific level, an inferred relationship between both morphometrics and Mg/Ca ratios and feeding habits seems to exist, as was stated in previous studies (e.g., Contreras & Castilla, 1987; Hagen, 2008; Lawrence & Jangoux, 2013). *A. lixula* shows a tendency to carnivory and is clearly separated from the three herbivores in the PCA and HCPC (Table 2, Figure 10). This species presents the most elongated teeth and longest demi‐pyramids in relation to test diameter (Table 1). *P. lividus* is characterized as an omnivore generalist with a tendency to herbivory, which is depicted in the PCA results (Figure 10). Finally, *D. setosum* and *S. granularis* appear to have similar feeding preferences, feeding primarily on coralline algae (Table 2). Both species also exhibited similar intraspecific trends, changing their demi‐pyramid elongation index, while also presenting the highest Mg/Ca ratios (Figure 4, Table 1). Higher Mg/Ca ratios might be a result of a Mg‐rich diet, as previously shown (Kolbuk et al., 2019). The similarities between *S. granularis* and *D. setosum* are most prominent when observing the clusters produced by HCPC, where *S. granularis* shares the same cluster with *D. setosum* (Figure 10).

There is evidence that another representative of the genus Diadema, namely *Diadema africanum*, can restrict the distribution of *S. granularis* in the Canaries archipelago (Tuya et al., 2001, 2007). It was also found that *D. africanum* can have detrimental effects for the densities of *S. granularis* in Madeira archipelago (Lourenço et al., 2022). The results presented here indicate that *D. setosum* might share the same feeding habits with *S. granularis* in the Eastern Mediterranean Sea, either leading to competition or additional pressure on calcareous algae when these species cooccur.

## 5 CONCLUSION

This study integrates mineralogical and morphometric data to infer relationships between feeding habits and various tooth features at both taxonomic and population levels within the Eastern Mediterranean echinoid communities. Through the examination of the Mg/Ca ratio alongside morphological characteristics, insights are gained as to how the structure of the tooth is tailored to fulfill specific ecological roles. This approach has provided valuable insights into the ecological roles of each species and their responses to environmental factors. Moving forward, this multidisciplinary approach can serve as a model for future studies seeking to unravel complex ecological dynamics within marine ecosystems.

## AUTHOR CONTRIBUTIONS

**Dimitris Vafidis:** Conceptualization (lead); formal analysis (supporting); project administration (lead); supervision (lead); visualization (lead); writing – original draft (lead); writing – review and editing (lead). **Anastasios Varkoulis:** Conceptualization (lead); data curation (lead); formal analysis (lead); methodology (lead); software (lead); visualization (lead); writing – original draft (lead); writing – review and editing (lead). **Stefanos Zaoutsos:** Data curation (supporting); methodology (equal). **Konstantinos Voulgaris:** Conceptualization (lead); methodology (lead); software (lead); visualization (lead); writing – original draft (lead); writing – review and editing (lead).

## FUNDING INFORMATION

This research was not funded.

## CONFLICT OF INTEREST STATEMENT

The authors declare no conflicts of interest.

## Data Availability

The data used for this manuscript are uploaded to Dryad: https://doi.org/10.5061/dryad.7m0cfxq0c.

## References

[ece311251-bib-0001] Agnetta, D. , Bonaviri, C. , Badalamenti, F. , Scianna, C. , Vizzini, S. , & Gianguzza, P. (2013). Functional traits of two co‐occurring sea urchins across a barren/forest patch system. Journal of Sea Research, 76, 170–177.

[ece311251-bib-0002] Androulidakis, Y. S. , & Krestenitis, Y. N. (2022). Sea surface temperature variability and marine heat waves over the Aegean, Ionian, and Cretan seas from 2008–2021. Journal of Marine Science and Engineering, 10(1), 42.

[ece311251-bib-0003] Azzurro, E. , Milazzo, M. , Maynou, F. , Abelló, P. , & Temraz, T. (2011). First record of *Percnon gibbesi* (H. Milne Edwards, 1853) (Crustacea: Decapoda: Percnidae) from Egyptian waters. Aquatic Invasions, 5, S123–S125.

[ece311251-bib-0004] Bianchi, C. N. (2007). Biodiversity issues for the forthcoming tropical Mediterranean Sea. Biodiversity in Enclosed Seas and Artificial Marine Habitats, 193, 7–21.

[ece311251-bib-0005] Black, R. , Johnson, M. S. , & Trendall, J. T. (1982). Relative size of Aristotle's lantern in *Echinometra mathaei* occurring at different densities. Marine Biology, 71, 101–106.

[ece311251-bib-0006] Boada, J. , Arthur, R. , Alonso, D. , Pagès, J. F. , Pessarrodona, A. , & Oliva, S. (2017). Immanent conditions determine imminent collapses: Nutrient regimes define the resilience of macroalgal communities. Proceedings of the Royal Society B: Biological Sciences, 284, 20162814. 10.1098/rspb.2016.2814 PMC5378086 28330920

[ece311251-bib-0007] Bonaviri, C. , Vega Fernández, T. , Fanelli, G. , Badalamenti, F. , & Gianguzza, P. (2011). Leading role of sea urchin *Arbacia lixula* in maintaining barren state in southwestern Mediterranean. Marine Biology (Heidelberg, Germany), 158, 2505–2513.

[ece311251-bib-0011] Cabral de Oliveira, M. (1991). Survival of seaweeds ingested by three species of tropical sea urchins from Brazil. Hydrobiologia, 222, 13–17.

[ece311251-bib-0013] Contreras, S. , & Castilla, J. C. (1987). Feeding behaviour and morphological adaptations in two sympatric sea urchins in central Chile. Marine Ecology Progress Series, 38, 217–224.

[ece311251-bib-0014] Coppard, S. E. , & Cambell, A. C. (2007). Grazing preferences of diadematid echinoids in Fiji. Aquatic Botany, 86, 204–212.

[ece311251-bib-0015] Coppard, S. E. , & Campbell, A. C. (2006). Taxonomic significance of test morphology in the echinoid genera Diadema Gray, 1825 and Echinothrix Peters,1853 (Echinodermata). Zoosystema, 28, 93–112.

[ece311251-bib-0016] De Riddler, C. M. , & Lawrence, J. M. (1982). Food and feeding mechanisms: Echinoidea. In M. Jangoux & J. M. Lawrence (Eds.), Echinoderm nutrition (pp. 57–115). Balkema.

[ece311251-bib-0017] deVries, M. S. , Webb, S. J. , & Taylor, J. R. A. (2019). Re‐examination of the effects of food abundance on jaw plasticity in purple sea urchins. Marine Biology, 166, 14.

[ece311251-bib-0018] Dumont, C. P. , Lau, D. C. C. , Astudillo, J. C. , Fong, K. F. , Chak, S. T. C. , & Qiu, J. W. (2013). Coral bioerosion by the sea urchin *Diadema setosum* in Hong Kong: Susceptibility of different coral species. Journal of Experimental Marine Biology and Ecology, 441, 71–79.

[ece311251-bib-0019] Ebert, T. A. (1980). Relative growth of sea urchin jaws: An example of plastic resource allocation. Bulletin of Marine Science, 30, 467–474.

[ece311251-bib-0020] Ebert, T. A. , Hernández, J. C. , & Clemente, S. (2014). Annual reversible plasticity of feeding structures: Cyclical changes of jaw allometry in a sea urchin. Proceedings of the Royal Society B: Biological Sciences, 281, 20132284. 10.1098/rspb.2013.2284 PMC3924062 24500161

[ece311251-bib-0021] Epherra, L. , Crespi‐Abril, A. M. , Pablo, E. , Cledón, M. M. , Enrique, M. , & Rubilar, T. (2015). Morphological plasticity in the Aristotle's lantern of *Arbacia dufresnii* (Phymosomatoida: Arbaciidae) of the Patagonian coast. Revista de Biología Tropical, 63(Suppl. 2), 339–351.

[ece311251-bib-0022] Espinosa, H. D. , Zaheri, A. , Nguyen, H. , Restrepo, D. , Daly, M. , Frank, M. , & McKittrick, J. (2019). In situ Wear study reveals role of microstructure on self‐sharpening mechanism in sea urchin teeth. Matter, 1(5), 1246–1261.

[ece311251-bib-0077] González‐Durán, E. , Castell, J. D. , Robinson, S. M. C. , & Blair, J. T. (2008). Effect of dietary lipids on the fatty acid composition and lipid metabolism of the green sea urchins Strongylocentrotus droebachiensis. Aquaculture, 276, 120–129.

[ece311251-bib-0023] Gorzelak, P. , Dery, A. , Dubois, P. , & Stolarski, J. (2017). Sea urchin growth dynamics at microstructural length scale revealed by Mn‐labeling and cathodoluminescence imaging. Frontiers in Zoology, 14, 42. 28855950 10.1186/s12983-017-0227-8 PMC5574115

[ece311251-bib-0025] Hagen, N. T. (2008). Enlarged lantern size in similar‐sized, sympatric, sibling species of Strongylocentrotid sea urchins: From phenotypic accommodation to functional adaptation for durophagy. Marine Biology, 153, 907–924.

[ece311251-bib-0026] Hermans, J. , Borremans, C. , Willenz, P. , André, L. , & Dubois, P. (2010). Temperature, salinity and growth rate dependences of Mg/Ca and Sr/Ca ratios of the skeleton of the sea urchin *Paracentrotus lividus* (Lamarck): An experimental approach. Marine Biology, 157, 1293–1300.

[ece311251-bib-0027] Hill, S. K. , & Lawrence, J. M. (2003). Habitats and characteristics of the sea urchins *Lytechinus variegatus* and *Arbacia punctulata* (Echinodermata) on the Florida gulf‐coast shelf. Marine Ecology (Berlin, Germany), 24, 15–30.

[ece311251-bib-0028] Hipolito, C. , Neto, R. M. , Casto, T. , Dionisio, M. A. , Prestes, A. C. , Azevedo, J. , Martins, G. M. , & Neto, A. I. (2020). Frondose and turf‐dominated marine habitats support distinct trophic pathways: Evidence from 15N and 13C stable isotope analyses. Arquipelago – Life and Marine Sciences, 37, 37–44.

[ece311251-bib-0029] Husson, F. , Josse, J. , & Pages, J. (2010). Principal component Methods‐hierarchical clustering‐Partitional clustering: Why would we need to choose for visualizing data. Agrocampus Ouest.

[ece311251-bib-0032] Johnson, C. R. , & Mann, K. H. (1982). Adaptations of *Strongylocentrotus droebachiensis* for survival on barren grounds in Nova Scotia. In J. M. Lawerence (Ed.), International echinoderms conference, Tampa Bay (pp. 277–283). Balkema.

[ece311251-bib-0035] Killian, C. E. , Metzler, R. A. , Gong, Y. , Churchill, T. H. , Olson, I. C. , Trubetskoy, V. , Christensen, M. B. , Fournelle, J. H. , Carlo, F. D. , Cohen, S. , Mahamid, J. , Scholl, A. , Young, A. , Doran, A. , Wilt, F. H. , Coppersmith, S. N. , & Gilbert, P. U. P. A. (2011). Selfsharpening mechanism of the sea urchin tooth. Advanced Functional Materials, 21(4), 682–690.

[ece311251-bib-0036] Kolbuk, D. , Dubois, P. , Stolarski, J. , & Gorzelak, P. (2019). Effects of seawater chemistry (Mg2+/Ca2+ ratio) and diet on the skeletal Mg/Ca ratio in the common sea urchin *Paracentrotus lividus* . Marine Environmental Research, 145, 22–26. 30777345 10.1016/j.marenvres.2019.02.006

[ece311251-bib-0037] Kougioumoutzis, K. , Valli, A. T. , Georgopoulou, E. , Simaiakis, S. M. , Triantis, K. A. , & Trigas, P. (2016). Network biogeography of a complex Island system: The Aegean archipelago revisited. Journal of Biogeography, 44, 651–660.

[ece311251-bib-0040] Lawrence, J. M. , & Jangoux, M. (2013). Cidaroids. In J. M. Lawrence (Ed.), Sea urchins: Biology and ecology (pp. 225–242). Academic Press.

[ece311251-bib-0041] Levitan, D. R. (1991). Skeletal changes in the test and jaws of the sea urchin *Diadema antillarum* in response to food limitation. Marine Biology, 111, 431–435.

[ece311251-bib-0042] Lourenço, S. , José, R. , Neves, P. , Góis, A. , Cordeiro, N. , Andrade, C. , & Ribeiro, C. (2022). Population density, reproduction cycle and nutritional value of *Sphaerechinus granularis* (Echinodermata: Echinoidea) in an oceanic insular ecosystem. Frontiers in Marine Science, 8, 699942.

[ece311251-bib-0043] Ma, Y. , Aichmayer, B. , Paris, O. , Fratzl, P. , Meibom, A. , Metzler, R. A. , Politi, Y. , Addadi, L. , Gilbert, P. U. P. A. , & Weiner, S. (2009). The grinding tip of the sea urchin tooth exhibits exquisite control over calcite crystal orientation and Mg distribution. Proceedings of the National Academy of Sciences of the United States of America, 106(15), 6048–6053. 19332795 10.1073/pnas.0810300106 PMC2662956

[ece311251-bib-0044] Ma, Y. R. , Weiner, S. , & Addadi, L. (2007). Mineral deposition and crystal growth in thecontinuously forming teeth of sea urchins. Advanced Functional Materials, 17, 2693–2700.

[ece311251-bib-0045] Märkel, K. (1970). The tooth skeleton of *Echinometra mathaei* (Blainville) (Echinodermata, Echinoidea). Annotationes Zoologicae Japonenses, 43, 188–199.

[ece311251-bib-0046] Markel, K. , & Gorny, P. (1973). Zur funktionellen anatomieder seeigelzahne (Echinodermata, Echinoidea). Zeitschrift für Morphologie und Ökologie der Tiere, 75, 223–242.

[ece311251-bib-0048] Masic, A. , & Weaver, J. (2015). Large area sub‐micron chemical imaging of magnesium in sea urchin teeth. Journal of Structural Biology, 189, 269–275. 25557499 10.1016/j.jsb.2014.12.005

[ece311251-bib-0049] McClanahan, T. R. , & Muthiga, N. A. (1988). Changes in Kenyan coral reef community structure and function due to exploitation. Hydrobiologia, 166, 269–276.

[ece311251-bib-0078] Minitab, LLC . (2019). Minitab. https://www.minitab.com

[ece311251-bib-0053] Pancucci‐Papadopoulou, M. A. , Raitsos, D. E. , & Corsini‐Foka, M. (2012). Biological invasions and climatic warming: Implications for south eastern Aegean ecosystem functioning. Journal of the Marine Biological Association of the United Kingdom, 92(4), 777–789.

[ece311251-bib-0079] Petihakis, G. , Triantafyllou, G. , Pollani, A. , Koliou, A. , & Theodorou, A. (2005). Field data analysis and application of a complex water column biogeochemical model indifferent areas of a semi‐enclosed basin: towards the development of an ecosystem management tool. Marine environmental research, 59, 493–518. 15603771 10.1016/j.marenvres.2004.07.004

[ece311251-bib-0054] Prado, P. , Alcoverro, T. , & Romero, J. (2010). Influence of nutrients in the feeding ecology of seagrass (*Posidonia oceanica* L.) consumers: A stable isotopes approach. Marine Biology (Heidelberg, Germany), 157, 715–724.

[ece311251-bib-0055] Privitera, D. , Chiantore, M. , Mangialajo, L. , Glavic, N. , Kozul, W. , & Cattaneo‐Vietti, R. (2008). Interand intra‐specific competition between *Paracentrotus lividus* and *Arbacia lixula* in resourcelimited barren areas. Journal of Sea Research, 60(3), 184–192.

[ece311251-bib-0056] Qiu, J. W. , Lau, D. C. , Cheang, C. C. , & Chow, W. K. (2014). Community‐level destruction of hard corals by the sea urchin *Diadema setosum* . Marine Pollution Bulletin, 85, 783–788. 24360335 10.1016/j.marpolbul.2013.12.012

[ece311251-bib-0058] Sartoretto, S. , & Francour, P. (1997). Quantification of bioerosion by *Sphaerechinus granularis* on ‘Coralugène’ concretions of the Western Mediterranean. Journal of the Marine Biological Association of the UK, 77, 565–568.

[ece311251-bib-0060] Smith, A. M. , Clark, D. E. , Lamare, M. D. , Winter, D. J. , & Byrne, M. (2016). Risk and resilience: Variations in magnesium in echinoid skeletal calcite. Marine Ecology Progress Series, 561, 1–16.

[ece311251-bib-0061] Smith, L. D. (2009). The role of phenotypic plasticity in marine biological invasions. In G. Rilov & J. A. Crooks (Eds.), Biological invasions in marine ecosystems. Ecological studies (Vol. 204, pp. 177–202). Springer.

[ece311251-bib-0064] Trenzado, C. E. , Hidalgo, F. , Villanueva, D. , Furn'e, M. , Díaz‐Casado, M. E. , Merino, R. , & Sanz, A. (2012). Study of the enzymatic digestive profile in three species of Mediterranean sea urchins. Aquaculture, 344, 174–180.

[ece311251-bib-0065] Tuya, F. , Cisneros‐Aguirre, J. , Ortega‐Borges, L. , & Haroun, R. J. (2007). Bathymetric segregation of sea urchins on reefs of the canarian archipelago: Role of flow‐induced forces. Estuarine, Coastal and Shelf Science, 73, 481–488.

[ece311251-bib-0066] Tuya, F. , Martín, J. A. , Reuss, G. M. , & Luque, A. (2001). Food preferences of the sea urchin *Diadema antillarum* in gran Canaria (Canary Islands, central‐east Atlantic Ocean). Journal of the Marine Biological Association of the UK, 81, 845–849.

[ece311251-bib-0067] Vafidis, D. , Antoniadou, C. , Voulgaris, K. , Varkoulis, A. , & Apostologamvrou, C. (2021). Abundance and population characteristics of the invasive sea urchin *Diadema setosum* (Leske, 1778) in the south Aegean Sea (eastern Mediterranean). Journal of Biological Research‐Thessaloniki, 28, 11. 10.1186/s40709-021-00142-9 PMC8138991 34016180

[ece311251-bib-0068] Varkoulis, A. , Voulgaris, K. , Zaoutsos, S. , Stratakis, A. , & Vafidis, D. (2020). Chemical composition and microstructural morphology of spines and tests of three Common Sea urchins species of the sublittoral zone of the Mediterranean Sea. Animals, 10(8), 1351. 32759777 10.3390/ani10081351 PMC7460165

[ece311251-bib-0069] Verlaque, M. (1981). Preliminary data on some Posidoniafeeders. Rapports et Procès‐Verbaux des Réunions Commission Internationale Pour l'exploration Scientifique de la Mer Méditerranée, 27, 201–202.

[ece311251-bib-0070] Voulgaris, K. , Varkoulis, A. , Zaoutsos, S. , Stratakis, A. , & Vafidis, D. (2021). Mechanical defensive adaptations of three Mediterranean Sea urchin species. Ecology and Evolution, 11, 17734–17743. 35003635 10.1002/ece3.8247 PMC8717311

[ece311251-bib-0071] Wang, R. Z. , Addadi, L. , & Weiner, S. (1997). Design strategies of sea urchin teeth: Structure, composition and micromechanical relations to function. Philosophical Transactions of the Royal Society of London. Series B, Biological Sciences, 352, 469–480. 9163824 10.1098/rstb.1997.0034 PMC1691937

[ece311251-bib-0072] Wangensteen, O. S. , Turon, X. , García‐Cisneros, A. , Recasens, M. , Romero, J. , & Palacín, C. (2011). A wolf in sheep's clothing: Carnivory in dominant sea urchins in the Mediterranean. Marine Ecology Progress Series, 441, 117–128.

[ece311251-bib-0074] Yokes, B. , & Galil, B. S. (2006). The first record of the needle‐spined urchin *Diadema setosum* (Leske, 1778) (Echinodermata: Echinoidea: Diadematidae) from the Mediterranean Sea. Aquatic Invasions, 3, 188–190.

[ece311251-bib-0075] Zervakis, V. , Georgopoulos, D. , & Drakopoulos, P. G. (2000). The role of the North Aegean in triggering the recent eastern Mediterranean climatic changes. Journal of Geophysical Research, 105(C11), 26103–26116.

[ece311251-bib-0076] Ziegler, A. , Stock, S. R. , Menze, B. H. , & Smith, A. B. (2012). Macro‐ and microstructural diversity of sea urchin teeth revealed by large‐scale mircro‐computed tomography survey. Proceedings of SPIE, 8506, 85061G.

